# Association between hereditary predisposition to common cancers and congenital multimalformations

**DOI:** 10.1111/cga.12329

**Published:** 2019-03-12

**Authors:** Fabrice Kwiatkowski, Isabelle Perthus, Nancy Uhrhammer, Christine Francannet, Marie Arbre, Yannick Bidet, Yves‐Jean Bignon

**Affiliations:** ^1^ Oncogenetics Department Centre Jean Perri (Comprehensive Cancer Center) Clermont‐Ferrand France; ^2^ Laboratory of Mathematics: Probabilities and Applied Statistics Clermont‐Auvergne University Clermont‐Ferrand France; ^3^ Medical Genetics Department Study Center of Congenital Malformations in Auvergne (Centre d'Etude des Malformations Congénitales en Auvergne) Clermont‐Ferrand France

**Keywords:** BRCA, cancer syndrome, congenital malformation, HBOC, oncogenetics

## Abstract

In a previous article we reported that mutations favoring cancer at adulthood seemed to improve fertility and limit miscarriages. Because spontaneous abortion may result from anomalies in embryo, we questioned if an increased frequency of congenital malformation could be evidenced among cancer‐prone families. Oncogenetics database (≈193 000 members) of the comprehensive cancer center Jean Perrin was crossed with regional registry of congenital malformations (≈10 000). Among children born between 1986 and 2011, 176 children with malformation matched in both databases. In breast/ovaries cancer‐prone families, the risk for malformations was multiplied by 2.4 [1.2‐4.5] in case of a BRCA1 mutation. Frequencies of malformation in BRCA2 and MMR mutated families were similar to families without a cancer syndrome. In comparison to malformations concerning a unique anatomical system, multimalformations were significantly more frequent in case of BRCA or MMR mutations: compared to families without cancer syndrome, the risk of multimalformations was multiplied by 4.1 [0.8‐21.7] for cancer‐prone families but with no known deleterious mutation, by 6.9 [1.2‐38.6] in families with a known mutation but an unknown parental mutational status and by 10.4 [2.3‐46.0] when one parent carried the familial mutation. No association with the type of anatomical system was found, nor with multiple births. These results suggest that BRCA and MMR genes play an important role in human embryogenesis and that if their function is lowered because of heterozygote mutations, congenital malformations are either more likely (BRCA1 mutations) and/or more susceptible to concern several anatomical systems.

## 1 INTRODUCTION

Predisposition to cancer in early adulthood exerts selective pressure on predisposed individuals by reducing life expectancy and consequently the length of the reproductive period. Mutations in one of the BRCA genes, with an associated risk of cancer starting as early as age 30, are relatively frequent in spite of this selective pressure. The persistence of such mutations in the population is demonstrated with founder mutations known to be thousands of years old.^1^, ^2^, ^3^ In a large retrospective survey of our oncogenetic database, we found that in families predisposed to breast/ovarian cancer, women from BRCA mutated families had a 36% lower miscarriage frequency (*P* = 0.015) than those from families with no known deleterious mutation; among individuals of both genders tested for a BRCA mutation, childless individuals were 22% less frequent for carriers (*P* = 0.0022) and the interval between first and last child was 16% longer (*P* = 0.042).^4^, ^5^ Although the underlying biological mechanisms are not yet known, this finding could suggest two opposing hypotheses:

1. Natural miscarriage triggers could be inhibited by defective genetic pathways that predispose to cancer. Considering that about 50%‐70% of miscarriages are caused by cytogenetic abnormalities^4^, ^5^, ^6^, ^7^, ^8^ of which a majority are induced by de novo aneuploidy,^9^ a higher frequency of congenital anomalies might be seen among carriers of mutations in these pathways.
2. Mutations predisposing to cancer at adulthood could reduce the risk for embryonic or fetal malformation—by an unknown genetic mechanism—and thus diminish the resulting frequency of miscarriage in the mutated population.

To the best of our knowledge, no research has been published on the incidence of congenital malformations in the offspring of parents with hereditary cancer risk. Sources of data on both conditions were available in the Auvergne region of France and were extensive enough to enable research regarding this issue. We crossed the information available in two large databases, the first used by the oncogenetic service of Centre Jean Perrin Comprehensive Cancer Center containing pedigrees of cancer‐prone families and the second from the regional register of congenital malformations that includes all children born with a congenital malformation. The study period corresponded to 26 years, from January 1986 to December 2011.

## 2 MATERIALS AND METHODS

The Auvergne region contains about 2% of the national population.^10^ Because of its distance from ports and major trade routes and its traditional rural character, migration inflows have always been limited. This makes Auvergne a good model for long‐term oncogenetic studies.

### 2.1 Regional registry of congenital abnormalities

The Centre d'Etude des Malformations Congénitales en Auvergne (CEMC‐Auvergne) is one of seven regional registries of congenital malformations in France. Launched in 1983, it is certified by the National Committee of Registries. It concerns all mothers giving birth in either public or private clinics in the region, about 14 000 births per year, including stillbirths and therapeutic abortions. All malformed newborns are registered as they are born after a pregnancy of at least 22 weeks of amenorrhea or if pregnancy was interrupted for congenital malformation regardless of the duration of amenorrhea. For livebirths, the diagnosis of malformation must be made during the first year of life. Exhaustivity is ensured because each health institution must declare cases of malformation and more than 99% of women give birth in public or private maternities.

All types of malformations are registered, including single or associated malformations, multiple syndromes (identified or not), with or without abnormal karyotype. Excluded are inborn errors of metabolism and minor deformations (hip shift without luxation, foot deformation, angioma or naevi smaller than 4 cm^2^, umbilical hernia with no need for surgery). Statistical analyses were performed either using the four categories of malformation (unique, multiple, syndromal, and karyotypic) or using only two groups, unique malformations vs all other three categories that are more extended.

We selected the 10 026 livebirth cases in the registry. Considering about 364 000 children were born in Auvergne during the 25‐year study period, this corresponds to a malformation frequency of 2.8%, a rate compatible to the nation‐wide estimation of 2.4% in 2011 to 2012.^11^

### 2.2 Centre Jean Perrin Oncogenetics database

Created in 1988, the oncogenetics department of the regional comprehensive anticancer Centre Jean Perrin is the only center in the region offering evaluation of hereditary cancer risk. A large majority of local individuals belonging to high‐risk families seeking oncogenetics advice address this department. At the time of this study, the database included about 190 000 family members from 6600 families. Based on the population size of Auvergne and the expected prevalence of women with BRCA mutations in France, about 45% of women with familial breast/ovarian cancer risk in the region are included in the database.

Types of cancer risk were grouped by geneticists into several categories. It was based on their expertise in the early days and later on the calculation of scores like Eisinger^12^ or Manchester^13^ for hereditary breast and ovarian syndrome or, for hereditary colon cancer syndrome (HNPCC), using Amsterdam^14^ and/or Bethesda criteria.^15^ Breast/ovarian families were divided into families with BRCA1 or BRCA2 mutations and families with breast/ovarian cancer predisposition but no diagnosed BRCA mutation. PALB2 mutations could not be studied because of the too recent discovery of the implication of this gene in HBOC. Lynch syndrome families were grouped with colon cancer syndrome and again split into two classes: HNPCC/colon syndrome either with or without a known mutation in the mismatch repair genes (MLH1, MSH2, MSH6, or PMS2). When a family was diagnosed with both breast/ovarian and colon syndromes, it was placed within the group corresponding to the first indication given by the oncogeneticist. All other cancer risks (prostate, kidney, thyroid, hematological, digestive tract other than HNPCC/colon) were excluded from our analysis because of the wide heterogeneity of syndromes. A reference group included all individuals and their family members who consulted at the oncogenetics department but for who no cancer risk was diagnosed and therefore no genetic test was performed. A priori, this group was not exposed to a higher cancer risk than the general population, and was assumed to carry the same congenital malformation risk. Another control group was composed of individuals testing negative for a known familial mutation.

### 2.3 Database matching

**Figure 1:**
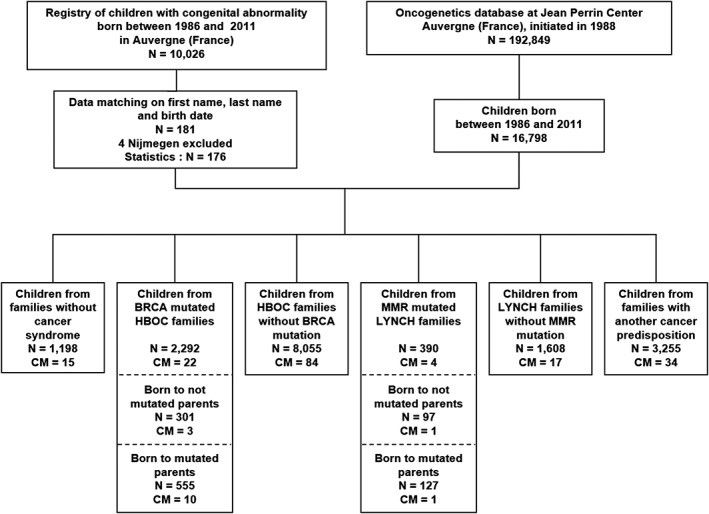
Data‐matching flowchart of the regional registry of congenital malformations and CJP oncogenetics database (CM, congenital malformation; HBOC, hereditary breast/ovarian cancer; Lynch, Lynch syndrome)

A temporary mixed database was constituted to evaluate the frequencies of congenital abnormalities: children born alive between 1986 and 2011, regardless of parental mutation status and type of cancer predisposition were extracted from our oncogenetic database. A computer “robot” was developed to match its records with those of the malformations register, based on similar/close children names, date of birth, mother's names, and age of parents; a visual control list permitted to validated manually each proposed match. To limit bias, that is, artificially increase the risk for malformation in particular subgroups of cancer‐prone families because of a syndrome combining both cancers and malformations, four cases of microcephaly were excluded from the analysis: these cases were referred for genetic analysis on the basis of specific congenital abnormalities suggestive of Nijmegen breakage syndrome (NBS), a syndrome that includes strong predisposition to lymphoid malignancy.^16^ Their corresponding families were thus excluded from the data‐matching diagram (Figure 1). Fanconi anemia was considered for exclusion, but none of our cancer‐prone families presenting with this syndrome had children born after 1985.

One hundred and seventy‐seven children with congenital anomalies corresponded to children in our oncogenetic database. The 33 abnormalities found for the 3244 children belonging to families with cancer predispositions other than Lynch and HBOC were excluded from the analysis because of the wide heterogeneity of this population.

NB. A public repository has been created in order to make data available. It contains several Excel sheets with first, an explanation on how are organized other sheets, second the results sheets and finally detailed information by subgroup. The address of the repository is: http://www.cjp.fr/fichiers/Anoccan_data.zip

### 2.4 Ethics

CEMC‐Auvergne is certified by the National Committee of Registries (2012‐2015) and the database was authorized by the French Authority for personal data protection (CNIL no 1387396). The consent signed by parents to enter this registry, allows the use of clinical data for research purpose. Centre Jean Perrin Database was declared by the CNIL correspondent under the number 1621407V0 on January first 2001. This certificate was renewed by CNIL on May 17th 2017 (no 2030983V 1). Counselees signed an informed consent that enables the use of data for research purpose. Another special authorization was requested because of the French regulation regarding the merge of databases coming from different entities (here CCC Jean Perrin and CEMC belonging to the Regional University Hospital). This authorization was granted by CEERES (national expertize committee for research, studies and evaluation regarding health) on March 15th 2018 (no TPS 37636) and then by CNIL on May 18th 2018 (DR 2018‐108) which permitted us to perform our study without any special information to families members about it. Finally, study ethics approval was obtained on 25 July 2018 (CECIC Rhône‐Alpes‐Auvergne, Grenoble, IRB 5921, file number CE‐CIC‐GREN‐17‐13).

### 2.5 Statistical analysis

Each family where a deleterious mutation was diagnosed yields three sets of children: those born to carriers of the familial mutation, those born to parents of unknown mutation status, and children born to non‐carriers. In the first set, 682 children were born to parents carrying the familial mutation (555 BRCA+ and 127 MMR+). The second set comprises 1602 children born to families with a known cancer predisposing mutation but parental carrier status was unknown. Finally, the 398 children born to known non‐carriers (301 BRCA‐ and 97 MMR‐) form a new control group, as their parents do not carry any known mutation favoring cancer. Congenital anomaly frequencies were compared for children of parents positive for a mutation or of unknown status belonging to a mutated family, vs other situations.

Because the risk for congenital malformation increases in cases of multiple births,^17^, ^18^, ^19^, ^20^, ^21^ the frequency of these events was computed per subgroup. These frequencies were compared to frequencies of congenital anomaly both to verify that our statistics were not biased because of this, and also to control that these frequencies were in accordance with national figures.

To compare proportions of children with abnormalities between subsets, *χ* ^2^ test were used, or Fisher's exact test if needed. 95% confidence intervals were calculated assuming the number of observed cases followed the Poisson distribution of rare events. Cochran‐Armitage test for trend was used to evaluate the proportion increase of syndromal, chromosomal, and multiple malformations across mutational groups. The relation between proportions of twins and congenital abnormalities were tested using standard Pearson correlation. R version 3.0 and SEM software^22^ were used for statistics and data‐management.

## 3 RESULTS

In the oncogenetic database, 16 798 children with a known date of birth were born between 1986 and 2011, of whom 2292 belonged to families with a BRCA mutation and 390 to families with an MMR mutation. The registry contained 11 234 cases diagnosed during the same period, with 10 026 corresponding to the selection criteria. Considering about 364 000 children were born in Auvergne during the 26‐year study period, this corresponds to a malformation rate of 2.8%, a rate compatible to the nation‐wide estimation equal to 2.4% in 2011 to 2012.^11^ Overall, 176 children with congenital anomalies corresponded to children in our oncogenetic database (the matching process is described in the material and methods section).

### 3.1 Frequencies of congenital malformation

Because most families recruited at our center consult for breast/ovarian cancer syndrome, 62% of children belonged to HBOC families, that is, exposed to Hereditary Breast or Ovarian Cancer susceptibility (Table 1). The remaining corresponded to Lynch families (12%), families without any cancer syndrome (7%), and families consulting for various other cancer syndromes (19%).

**Table 1:** Repartition of abnormalities according to cancer predisposition and parental or familial mutation status

Children's origin	Children number	Families	Malformations	Malf. rate (%)	95%‐CI Poisson
**Control group 1: from families where no hereditary cancer risk has been diagnosed**	**1198**	**291**	**15**	**1.25**	**[0.70%‐2.07%]**
**from family where a BRCA1 mutation has been diagnosed, including those born to mutated or non‐mutated parents**	**1353**	**228**	**15**	**1.11**	**[0.58%‐1.77%]**
from families with a BRCA1 mutation but unknow status of parents	858		4	0.47	[0.07%‐1.05%]
children fathered by a parent carrier of a BRCA1 mutation	324	138	8	2.47	[1.09%‐4.99%]
from family where a BRCA1 mutation but born to non‐mutated parents (ie not exposed to the familial risk)	171	70	3	1.75	[0.36%‐5.13%]
**from family where a BRCA2 mutation has been diagnosed, including those born to mutated or non‐mutated parents**	**961**	**178**	**7**	**0.73**	**[0.36%‐1.63%]**
from families with a BRCA2 mutation but unknow status of parents	592		5	0.84	[0.37%‐2.18%]
children with a parent carrier of a BRCA2 mutation	239	105	2	0.84	[0.10%‐3.02%]
from family where a BRCA2 mutation has been diagnosed but born to non‐mutated parents (ie not exposed to the familial risk)	130	51	0	0.00	[0.00%‐2.84%]
**from family at hereditary breast/ovarian cancer risk, but without any known deleterious mutation diagnosed**	**8062**	**1699**	**84**	**1.04**	**[0.83%‐1.29%]**
**from family with a Lynch syndrome where a MMR mutation has been diagnosed, including those born to mutated or non‐mutated parents**	**390**	**83**	**4**	**1.03**	**[0.28%‐2.63%]**
from families with a MMR mutation but an unknown status of parents	166		2	1.20	[0.15%‐4.35%]
children with a parent carrier of a MMR mutation	127	55	1	0.79	[0.02%‐4.39%]
from family where a MMR mutation has been diagnosed but born to non‐mutated parents (ie not exposed to the familial risk)	97	37	1	1.03	[0.03%‐5.74%]
**from family at hereditary colon cancer risk, but without any MMR mutation diagnosed in the family**	**1613**	**338**	**17**	**1.05**	**[0.61%‐1.69%]**
**Control group 2: total from families where a mutation has been diagnosed but born to non‐mutated parents**	**398**	**158**	**4**	**1.01**	**[0.27%‐2.57%]**
**Total from families where a mutation has been diagnosed and born to mutated parents**	**690**	**298**	**11**	**1.59**	**[0.80%‐2.85%]**
**Excluded group: Children not included because they belong to families with other cancer syndrome**	**3255**	**782**	**34**	**1.04**	**[0.72%‐1.46%]**
**TOTAL**	**16798**	**3599**	**176**	**1.05**	**[0.90%‐1.22%]**

**Figure 2:**
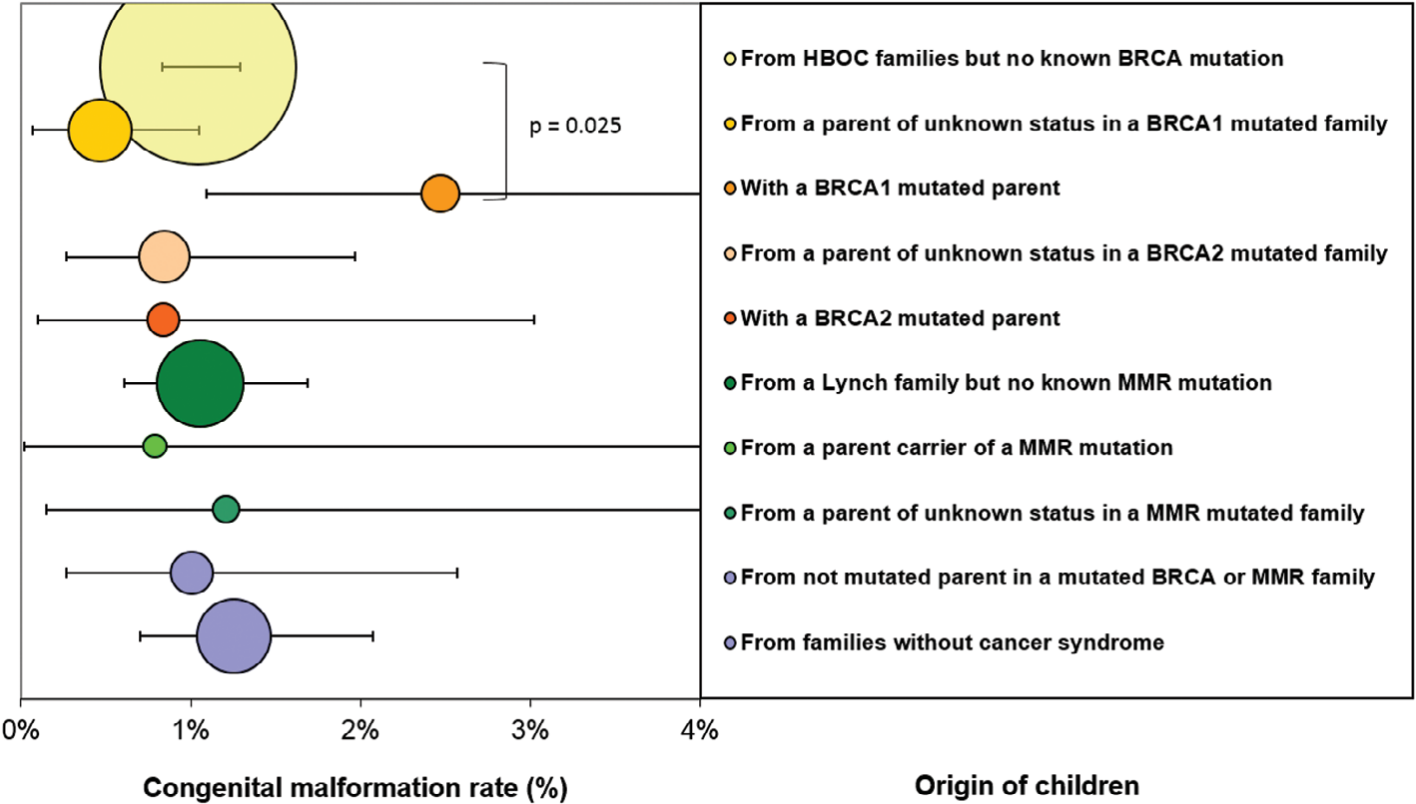
Frequency of congenital anomalies per sub‐group. Area of circles corresponds to group sample size. Error bars represent Poisson 95%‐CI of frequencies. The two bottom groups (no cancer syndrome and children born to non‐mutated parents) constitute the “normal” reference frequency

The overall malformation frequency was 1.05% [0.90‐1.22]. This frequency did not vary significantly according to the presence or the absence of familial cancer predisposition (1.03% vs 1.19% for both control groups together, *P* = 0.55) (Figure 2).

For children born to a BRCA1 mutated parent, the frequency of malformation (2.47%) was slightly higher than the frequency of both control groups (1.19%, *P* = 0.13), and when compared to the HBOC group without known deleterious mutation (1.04%), the risk for malformation was increased by RR = 2.37 [1.18‐4.78], *P* = 0.025. A comparison with all other groups together yielded the same risk increase: 2.42 [1.22‐4.81] (*P* = 0.025). This was not the case for BRCA2 (0.84%) or MMR mutations (0.79%).

### 3.2 Analysis according to categories of malformation

**Figure 3:**
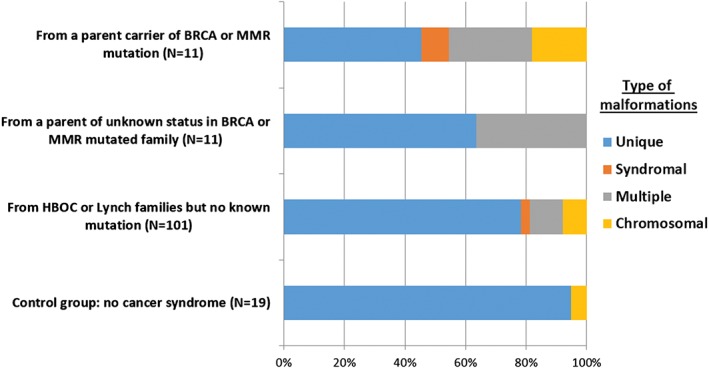
proportion of congenital anomalies per sub‐group according to anomaly type

Malformations were classified in four categories according to extent. The unique type (including single malformations or multiple malformations of a single organ) was the most frequent (75.6%), followed by multiple, that is, malformations concerning several organs (14.4%), chromosomal (7.8%), and syndromal (2.2%). These figures significantly differed from the repartition calculated over the whole registry, respectively 68.0%, 10.0%, 13.7%, and 8.3% (*P* = 0.00077), with an under‐representation of syndromal and chromosomal malformations in our families. Distribution varied significantly according to subgroups of cancer risk and parental mutation status (Figure 3).

If syndromal, multiple and chromosomal abnormalities were grouped together under the label “extended,” their proportion compared to unique malformations yield significant differences (*P* = 0.014) according to parental mutation status and a significant trend was objectivized (*P* = 0.0011) from the first to the fourth group (Table 2):

**Table 2:** Distribution of unique abnormalities vs syndromal, multiple and chromosomal abnormalities together (“Extended”) according to parental mutation status

Parental group	Malformations		Rate of extended malformation in malformed children			Number of children	Rate of extended malformation in all children		
	Unique	Extended	Rate (%)	RR	95%‐CI		Rate (%)	RR	95% CI
Control groups (no cancer syndrome)	18	1	5.3%	1		1596	0.06%	1	
No known mutation in the family	79	22	21.8%	4.1	[0.8 à 21.7]	9675	0.23%	3.6	[0.6‐23.5]
Unknown status in a mutated family	7	4	36.4%	6.9	[1.2 à 38.6]	1602	0.25%	4.0	[0.5‐30.2]
Parent a known mutation carrier	5	6	54.5%	10.4	[2.3 à 46.0]	682	0.88%	14.0	[2.8‐69.9]

Children of deleterious mutation carriers had a 10‐fold risk of “extended” malformation in comparison to the control group. A same trend was found if we only considered multi‐malformations (*P* = 0.0013). But no significant trend was found for syndromes alone (*P* = 0.13) and chromosomal anomalies (*P* = 0.18).

The frequencies of “extended” malformation per group increased proportionally to the probability that children might carry a deleterious mutation (ie, 100% divided by two in the group of carriers parents, 25% when the mutational status of the parent was unknown but from a mutated family, and so on): the proportion of “extended” malformations (Table 2) varied accordingly if it was calculated either over the total number of malformations or over the number of children per group (respectively *P* = 0.008 and 0.003). Meanwhile in these four groups, the global frequencies of malformation whatever the type (unique or “extended”) did not differ (*P* = 0.25).

Finally, as the risk of malformation tends to increase with father's age, due to an increased frequency of de novo mutations,^23^, ^24^ fathers' age was compared according to malformation types. Overall, no difference was found between the four groups of parents (*P* = 0.28) although the association between chromosomal malformations and older fathers was close to significance (*P* = 0.06). Mean fathers' age was respectively 31.2 ± 5.7 for unique malformations, multiple 31.4 ± 5.9, syndrome 31.3 ± 12.3, and chromosomal 36.7 ± 10.1. If age of fathers was split into two classes (<45 vs ≥45 years), the relative risk for chromosomal malformation with older fathers was multiplied by 9.6 [3.1‐29.4] (*P* = 0.01). Mean mothers' age was similar in all groups (*P* = 0.77), respectively 29.0 ± 5.0, 29.6 ± 4.6, 27.5 ± 3.1, and 29.9 ± 4.9.

### 3.3 Analysis according to anatomical system concerned by malformations

**Figure 4:**
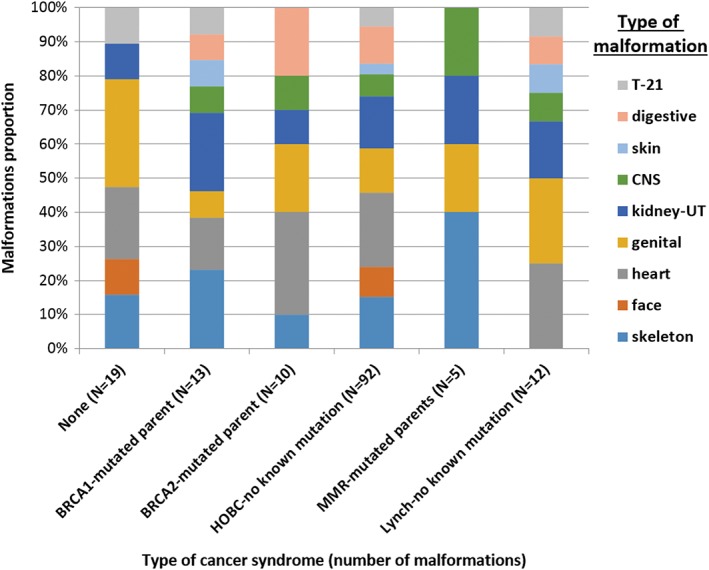
anatomical system concerned by malformation according to group of cancer risk (T‐21, Down syndrome; CNS, central nervous system; UT, urinary tract)

Malformations were reported for the following anatomical systems: heart in 33% of cases, skeleton 25%, genital 25%, digestive 14%, CNS 11%, face 10%, Down syndrome 9%, and skin 5% (Figure 4).

In our population, systems concerned by malformations did not significantly vary according to cancer risk groups (*P* ≈ 0.90).

### 3.4 Analysis of multiple births frequency

**Figure 5:**
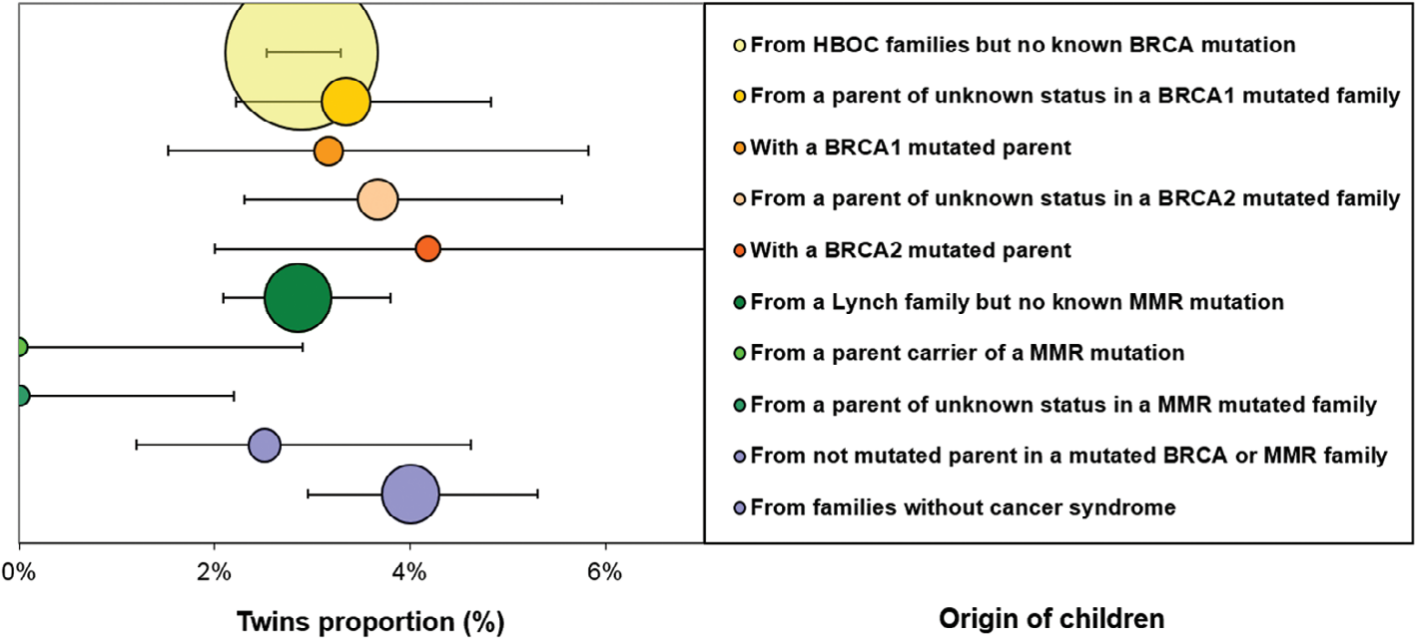
Proportion of twins among children registered in our study (error bars correspond to Poisson 95%CI)

Because associations have been described between multiple births and congenital malformation incidence, we investigated if frequencies of abnormalities and twinning were associated in our population. Two families registered triple births: they were grouped with twins. The overall frequency of multiple births was 2.47%. No association was found between frequencies of twins and frequencies of malformations (*r* = 0.07, *P* = 0.81). Seven malformations were reported among our 341 twins/triplets, all unique except one, multiple, malformations in one twin born to a BRCA2‐mutated parent. Five concerned heart anomalies and one skeletal anomaly, while the only multiple case associated heart, digestive and CNS malformations. Surprisingly, no twins were observed in families with MMR mutations: *P* = 0.00023 vs 3.38% of twins in the control families (Figure 5).

When all cancer syndromes together were compared to the control group, the frequencies of multiple births were similar (respectively 2.95% and 3.38%, *P* = 0.32). No interaction regarding congenital malformations was observed between multiple births and BRCA mutations (*P* = 0.89).

## 4 DISCUSSION

In our previous study,^4^ we observed fewer miscarriages in cancer‐prone families and suspected a possible increase of congenital malformations. In the present study, cancer predisposition was weakly associated with a higher incidence of congenital abnormalities in offspring. This association was entirely attributable to BRCA1 mutated parents who were associated with an increased risk of 2.37 [95% CI 1.18‐4.78] (*P* = 0.025) compared to HBOC families with no known BRCA mutation and 2.42 [1.22‐4.81] (*P* = 0.025) when compared to anyone. However, the level of evidence of this result is mild and the possibility that the higher frequency of congenital abnormalities in descendants of BRCA1‐mutated parents might be due to chance cannot be discarded. BRCA2 as well as MMR mutations did not seem to produce an increase in overall congenital malformation risk.

The main conclusion of our study concerns the type of congenital malformation: the incidence of multimalformations significantly increased with the probability of BRCA or MMR mutation in one of the parents. This trend argues in favor of a direct biological impact of these mutations on embryonic development. These genes are involved in DNA repair, and it is likely that when repair is less efficient, early genetic anomalies causing malformation may not be corrected and consequences concern several anatomical systems. This also suggests that some congenital malformations result from a reduced capacity of the embryo to repair DNA anomalies, whatever their cause (either spontaneous or related to an inherited anomaly). Such a hypothesis has been proposed in xeroderma pigmentosum in relation to disorders of DNA repair and transcription gene.^25^, ^26^ The association of these disorders and the risk for pre‐eclampsia^27^ is also interesting and confirms the relevance of our working hypothesis: mutations affecting DNA repair and transcription have an impact on both congenital malformations and miscarriage mechanisms.

The slight association of BRCA1 mutations with the risk for congenital malformation may be linked to the interaction between BRCA1 and the Notch signaling pathway. This interaction is a key regulator of breast cell differentiation^28^: dysregulations are associated with basal‐like tumors. Shifley et al reported the implication of this pathway in the development of the vertebral column in embryos and the occurrence of congenital skeletal defects when mutations disrupt the segmentation clock function controlled by Notch pathway.^29^ Although skeleton malformations were slightly more frequent (23%) when one parent carried a BRCA1 mutation than in other groups (15%), our study is not powered enough to confirm this hypothesis. In rodents, BRCA1 has proven to play an important role in the early development of embryos.^30^ Homozygous BRCA1 mutations are lethal: mutant mice die before 2 weeks of embryogenesis.^31^ Abnormalities often concern the neural tube, with 40% of the embryos presenting with varying degrees of spina bifida and anencephaly.^32^ For Hakem et al,^33^ the death of mutant embryos “*may be due to a failure of the proliferative burst required for the development of the different germ layers*.” In humans, no study of the impact of homozygote mutations on BRCA genes is available, likely because embryos are not viable. This is supported by a study of families where both parents carried a BRCA1 mutation^34^: because none of the children carried both mutated alleles, the authors concluded that the most likely reason was that BRCA homozygotes were not viable. Heterozygous mutations have been proposed to expose embryo to a lethal risk unless compensated by other particular genotypes, such as specific alleles of FMR1.^35^ This assertion was questioned in a study of Ashkenazi BRCA1/2 mutation carriers where FMR1 sub‐types were only slightly unbalanced.^36^ The hypothesis of possible genotypic compensations remains relevant and heterozygous mutations could very well favor multimalformations following a similar pattern as described in mice, but depending on a genomic context which remains to be defined.

BRCA2 interacts with a signaling pathway that regulates fibroblast growth factors, and mutations are associated with a wider variety of breast cancer phenotypes than BRCA1 mutations.^37^ Double heterozygosity involving BRCA1 and BRCA2 mutations have been reported in the literature,^38^ though was not more penetrant than BRCA1 mutation alone. It is unknown if double heterozygosity further increases the risk for congenital malformation.

MMR deficiencies have been associated to agenesis of corpus callosum and gray matter heterotopia^39^ and to neural tube defects.^40^ One of the three malformed children from MMR mutated families suffered from a CNS anomaly, while the others presented malformations of the kidneys and skeleton. This small population is of course insufficient to confirm any trend.

The rate of congenital abnormalities in Europe was determined in 2010 at 2.3% of all births, among which, 80% of newborns survive.^41^ More recent data estimated to be around 3% of livebirths and 15% to 20% of stillbirths in the state of Utah‐USA.^42^ Higher rates of congenital abnormalities have been reported in France: 3.3% in 2007 in Paris^43^ after an 85% increase from 1981 to 2007 of total abnormalities (stillbirths and livebirths together). For the period 1986 to 2011, an overall 2.8% malformation rate was estimated from Auvergne registry (including medical abortions for intra‐uterin malformations). French national estimations by National Health Institute (INVS) reported a 2.4% malformation rate for livebirths in 2011 to 2012.^11^ Our no cancer‐syndrome control group exhibited a 1.4% congenital abnormality frequency, suggesting that the database matching process was only able to flag about half of the expected congenital abnormalities for the children in the oncogenetics database. The origins of this discrepancy may include the way pedigrees are built and how they are updated. Oncogenetics mainly targets cancer in adulthood: for the most recent generation, dates of birth as well as the first and/or last names of children may be omitted. Secondly, probands are asked to update their pedigree when a new cancer is diagnosed, but not for the birth of a child. Therefore, many children born after the pedigree was built are missing. This lack is not related to the familial cancer risk and there is no reason why this would induce any bias in our results. But this limits the accuracy of our estimates and reduces the power of our study. Another reason could be responsible for this low frequency of malformations in our cohort: a younger age of mothers: mothers' age at their children birth was in average 29.5 ± 4.9 years and ranged between 16 and 59. These figures are in accordance with French national statistics (from 29.8 in 2007 to 30.7 in 2017).^43^ Finally, the frequency of syndromal and chromosomal malformations in our sample was low when compared to our registry (2% vs 8% in the registry for syndromes, *P* = 0.009 and 8% vs 14%, *P* = 0.023 for karyotype defects): indeed, genetic anomalies corresponding to labeled syndromes rarely induce cancer and those favoring cancer (Nijmegen for example) were excluded. This is also true for chromosomal anomalies (trisomy for example) that highly reduce the risk for solid tumors while it increases the risk for leukemia.^44^ Conversely multimalformations were more frequent in our sample (14% vs 10%, *P* = 0.05).

One source of bias may be related to the evolution in recent decades in prenatal diagnosis, where sensitivity of screening has strongly improved and malformations can be diagnosed earlier.^45^ For example, in our congenital malformation registry, the frequency of prenatal diagnosis among malformed children doubled from 26.4% before 2000 to 46.9% in recent years. Currently, the most severe malformations are ended by medical termination of pregnancy, while previously these pregnancies resulted in spontaneous abortion or stillbirth and would appear in the registry of congential malformations. Because we included only livebirths, we likely underestimated the frequency of severe malformation. To check if fetal deaths and/or medical abortions in recent years are correlated to BRCA mutations will be the subject of a further study.

Multiple births have been described to increase the risk for congenital anomalies^17^, ^18^, ^19^, ^20^, ^21^: the 3% twins’ frequency in our global population was equal to that cited by Boyle et al (2013) which confirms the fitness of our pedigree registration to expected figures. Overall, multiple births doubled the risk for congenital malformation. This increase was not significant (*P* = 0.12), but was similar to the risk of 1.71 [1.43‐2.12] found by Glinianaia et al^20^ in an English cohort. So, BRCA mutations did not seem to increase the risk for malformation in case of multiple births (*P* = 0.80), but our study was not powered to investigate this issue. We may however conclude that multiple births per category are not likely to bias our statistics. The only particularity found in our pedigrees is the absence of twins in the MMR families. The associated probability when compared to our reference group (*P* = 0.00023) let us suggest this might not be an artifact and MMR mutations may indeed interfere with the mechanisms favoring multiple births.

## 5 CONCLUSION

In our study, BRCA or MMR mutations significantly increased the risk for congenital multimalformations. This suggests that DNA repair genes play a role in embryonic development and that some congenital malformations may result from either less efficient repair, or from non‐repair functions of these genes. This agrees with the recent review on this issue by Terabayashi et al.^46^ This study partially confirms one of our working hypotheses: BRCA1 mutation carriers seem more likely to give birth to children with malformations. Further study is necessary to evaluate the influence of BRCA and MMR mutations on fetal deaths and/or medical abortions in case of fetal anomalies.

## DISCLOSURE OF INTEREST

None.
